# Severe Thrombocytopenic Purpura in a Child with Brucellosis: Case Presentation and Review of the Literature

**DOI:** 10.1155/2017/3416857

**Published:** 2017-01-03

**Authors:** Alexandros Makis, Aikaterini Perogiannaki, Nikolaos Chaliasos

**Affiliations:** Child Health Department, Faculty of Medicine, University of Ioannina, Ioannina, Greece

## Abstract

Brucellosis is still endemic and a significant public health problem in many Mediterranean countries, including Greece. It is a multisystemic disease with a broad spectrum of clinical manifestations including hematological disorders, such as anemia, pancytopenia, leucopenia, and thrombocytopenia. Thrombocytopenia is usually moderate and attributed to bone marrow suppression or hypersplenism. Rarely, autoimmune stimulation can cause severe thrombocytopenia with clinically significant hemorrhagic manifestations. We present the case of a girl with severe thrombocytopenic purpura as one of the presenting symptoms of* Brucella melitensis *infection. Treatment with intravenous immunoglobulin and the appropriate antimicrobial agents promptly resolved the thrombocyte counts. A review of similar published cases is also presented.

## 1. Introduction

Brucellosis is a zoonotic infection, which is endemic in Northwestern Greece [1] and is caused by Gram-negative bacteria that belong to the genus* Brucella*.* Brucella melitensis *is the most virulent form of the disease for humans. As far as the pediatric population is concerned, the infection is transmitted by direct contact with the contaminated animals or their fluids and by consumption of unpasteurized milk or other dairy products.

Several hematological manifestations such as anemia, leucopenia, thrombocytopenia, pancytopenia, leukocytosis, and thrombocytosis have been reported at diagnosis as well as during the course of the disease [2]. The pathophysiology of these abnormalities remains unclear but several mechanisms have been suggested including direct bone marrow infiltration, hypersplenism, hemophagocytosis, and activation of the immune system with production of autoantibodies.

Thrombocytopenia caused by* Brucella* is usually mild with no hemorrhagic complications and subsides after appropriate antibiotic treatment for the disease [1, 3]. Less often, immune mediated severe platelet depletion can occur at diagnosis or during the course of the disease, with significant bleeding manifestations. Isolated thrombocytopenia has been reported as the first symptom of the disease, initially misdiagnosed as primary immune thrombocytopenic purpura (ITP) [4]. The purpose of this paper is to describe the case of an immune mediated severe thrombocytopenia in a small child during the course of brucellosis and to present the relevant literature review.

## 2. Case Report

A 5.5-year-old girl presented with six-day fever up to 38.5°C with spikes mostly in the afternoon, accompanied by pain at the right wrist joint and right elbow joint. The girl's parents are stockbreeders and homemade dairy products had been offered to the child. Three days prior to her admission in our department, she was examined in another hospital, where no remarkable findings from the joints or other systems were noted. As shown in Table 1, laboratory tests were white blood cells 7.2 × 10^9^/L, platelets 180 × 10^9^/L, hemoglobin 131 g/L, erythrocyte sedimentation rate (ESR) 19 mm/h, C-reactive protein (CRP) 10 mg/L, monotest negative, alanine aminotransferase (AST) 62 U/L, positive Wright agglutination reaction (title 1/1280), and negative Rose Bengal. Based on the symptoms and the positive Wright reaction, treatment for brucellosis was initiated. Trimethoprim/sulfamethoxazole (TMP/SMX) (10 mg/kg/day) and rifampicin (15 mg/kg/day) were administered orally.

On the third day of treatment, generalized purpuric lesions were noted accompanied with severe thrombocytopenia (platelets 3 × 10^9^/L) and the child was referred to our hospital. On admission the child was febrile (38.8°C) and generalized petechial/purpuric rash, bruising, wet purpura on the palate, and gingival bleeding were noted, while the spleen and the liver were palpable 2 cm below the subcostal margin at the midclavicular line. Examination of the joints and the other systems did not reveal any pathological signs.

Laboratory tests showed white blood cells 7.5 × 10^9^/L (neutrophils 45%, lymphocytes 52%, and monocytes 3%), platelets 1 × 10^9^/L, hemoglobin 120 g/L, ESR 15 mm/h, CRP 7 mg/L, AST 64 U/L, urea 4.1 mmol/L, creatinine 92 *μ*mol/L, and total bilirubin 12 *μ*mol/L. Wright agglutination reaction was positive (title 1/1280), as well as the specific IgM, IgA antibodies against* Brucella melitensis*. The specific IgG antibodies were negative. Blood cultures were sterile. Direct and indirect Coombs were negative. The quantification of immunoglobulins IgG, IgM, and IgA and complements C3 and C4 was within normal range. Antinuclear antibodies, anti-double stranded DNA antibodies, and rheumatoid factor were negative, while the antiplatelet antibody test was positive. Clotting time and fibrinogen were normal (Table 1).

Due to the very low platelet count and the mucous membrane hemorrhage, treatment with intravenous immunoglobulin (IVIG) (1 gr/kg/day for two days) was initiated, combined with antibiotic therapy with amikacin intravenously (15 mg/kg/day for seven days) and oral rifampicin. TMP/SMX treatment was stopped due to the suspicion of drug-induced thrombocytopenia.

The patient responded with a gradual increase in the number of platelets up to 218 × 10^9^/L five days after treatment. At this stage, oral TMP/SMX administration was reinitiated, without any negative effect on the platelet count. On the contrary, a rise in platelet number up to 503 × 10^9^/L was observed. Fever resolved at the second day of treatment and no new bleeding manifestations appeared. The patient was discharged with rifampicin and TMP/SMX as maintenance therapy for a total six-week treatment. She completed the treatment without further complications and during the regular follow-up platelet counts were normal (Table 1).

## 3. Discussion

Thrombocytopenia is one of the hematological manifestations of brucellosis in children with a variable percentage of 5–40% in several studies [1–3, 5, 6]. The pathogenic pathways of* Brucella*-related thrombocytopenia have not been fully elucidated, with hypersplenism, hemophagocytosis, bone marrow suppression, and antiplatelet antibodies production being the most possible mechanisms. It is usually moderate without clinically significant bleeding problems. Less often, severe isolated thrombocytopenia occurs, possibly immune mediated, mimicking ITP (Table 2).

In Greece, several cases of severe thrombocytopenia have been described over the years. In 1998, Benecos et al. from our department reported a case of a boy who presented with severe thrombocytopenic purpura with petechiae, epistaxis, oral bleeding, and positive antiplatelet antibodies. He was initially treated as ITP with IVIG with good clinical and hematological response. A few days later prolonged fever occurred with persistent hepatomegaly and positive blood culture for* Brucella melitensis* and the child was successfully treated with streptomycin and TMP/SMX [7]. Tsirka et al. reported an 11-year-old boy with anemia, leukopenia, and severe thrombocytopenic purpura, who was treated as ITP with IVIG with an initial good response. Surprisingly, a few days later a blood culture was positive for* Brucella melitensis* and treatment with gentamicin, doxycycline, and rifampicin was successfully implemented [8]. Tsolia et al. presented 39 children diagnosed with brucellosis over a period of 15 years in central Greece, two of whom had severe thrombocytopenic purpura and received oral antibiotic therapy with a good outcome [3].

In Turkey brucellosis remains a major public health problem, with approximately 15000 cases per year. Pediatric patients represent 20–25% of all cases [9]. Authors from several areas of the country have reported cases of isolated severe thrombocytopenia as the presenting feature of brucellosis that responded well to appropriate antibiotic treatment combined with corticosteroids or IVIG. Fever was noticed in most cases, with positive blood culture for* Brucella* [10–13].

Apart from these case reports, large series of pediatric patients with brucellosis have been studied in Turkey with interesting findings regarding* Brucella*-related severe thrombocytopenia. Akbayram et al. reported five cases presenting with isolated thrombocytopenia out of 187 children (2.6%) diagnosed with brucellosis in one hospital in Turkey between 2004 and 2010. All patients had a complete recovery and their platelet counts returned to normal after the administration of a combination of doxycycline and rifampicin or TMP/SMX and rifampicin [14]. During the same period in another hospital, Citak et al. retrospectively assessed the records of 146 children with brucellosis. Among them, 5 (3.4%) presented with immune thrombocytopenia, which was severe and manifested with a variety of symptoms and positive blood cultures for the bacteria. Apart from the appropriate antibiotic treatment, IVIG was administered with excellent response [6]. Aypak et al. evaluated the hematological findings in 69 children who were diagnosed with brucellosis over a one-year period [2010-2011] and 15.9% of them presented with thrombocytopenia restored after antibiotic treatment without the need of corticosteroids or IVIG [2]. In a most recent study with large numbers of patients from Eastern Turkey, Karaman et al. presented the hematological findings of 622 children with brucellosis over a 6-year period. Isolated severe thrombocytopenia was found in 16 patients (2.5%), which resolved with antibiotics or IVIG [5].

In countries of the Middle East where brucellosis is a significant health problem, isolated severe thrombocytopenia has been reported as a presenting symptom in several cases. In a series of 115 children with brucellosis in Saudi Arabia, four of them had severe thrombocytopenia and proper oral antibiotic therapy led to quick recovery [15]. In another study, clinically significant thrombocytopenia was found in 5 children from a population of 110 children with brucellosis and it was successfully treated with antibiotics [16]. Benjamin described two cases of children with fever, thrombocytopenic purpura, and mucosal bleeding which resolved promptly with the initiation of antimicrobial therapy. Thrombocytopenia was attributed to peripheral destruction since large platelets in the peripheral blood and proliferation of megakaryocytes in the bone marrow were identified: an image resembling ITP [17]. In 2010, Farah et al. in Lebanon reported a case of severe thrombocytopenic purpura as the sole manifestation of brucellosis in an 8-year-old boy. Giant platelets were found in his peripheral blood smear and bone marrow aspiration showed elevated megakaryocytes with no evidence of hemophagocytosis. He was successfully treated with gentamicin, TMP/SMX, and IVIG [18]. In a similar case from Iran, with isolated* Brucella*-related thrombocytopenia, administration of rifampicin, TMP/SMX, and IVIG solved the problem [19], while in another case from Israel sole antibiotic treatment was given because the hemorrhagic features were mild [20].

As it is apparent from reports in the literature, it is necessary for the general pediatrician to consider brucellosis in the differential diagnosis of thrombocytopenia, particularly in endemic areas. Children with the initial diagnosis of ITP who do not respond to treatment or present with fever, arthralgia, or hepatosplenomegaly should be also investigated for brucellosis, especially when there is a history of nonpasteurized dairy products consumption, a family history of brucellosis, or a preceding trip in endemic countries.

As far as our case is concerned, we faced some difficulties which raised certain concerns. The patient was admitted with extremely low platelet count and bleeding lesions of the mucous membranes. Consequently, the risk of internal or intracranial bleeding and the need for immediate intervention were urgent. Therefore, IVIG and intravenous treatment with aminoglycoside were administered, which led to a rapid and significant increase in platelet count.

Another discussion topic is the possibility of the involvement of the drugs already administered to the patient in the induction of thrombocytopenia. Rifampicin is a drug that could cause thrombocytopenia, but it is usually mild and clinically insignificant, whereas TMP/SMX can occasionally cause severe drop in platelet count with significant bleeding. Therefore, we decided to stop TMP/SMX treatment. Given that the patient had to continue double antimicrobial treatment beyond the acute phase for a total of six weeks, it was decided to continue the use of rifampicin. To exclude possible drug-induced thrombocytopenia from TMP/SMX, the drug was reintroduced after the stabilization of the patient's condition and this had no effect on the platelet count.

In conclusion, in regions where brucellosis is endemic, it should be included in the differential diagnosis in cases of ITP. There should be an individualized approach to each patient but in any case the administration of IVIG or corticosteroids is of great significance, while the final and main resolution occurs with appropriate antimicrobial therapy.

## Figures and Tables

**Table 1:** Hematological parameters and treatment at admission and during the course of the disease.

Treatment	3 days before admission	Admission	Day 5	Day 10	Week 6
	TMP/SMX, rifampicin	IVIG, amikacin, rifampicin	TMP/SMX, rifampicin		
WBC (×10^9^/L)	7.2	7.5	8.7	7.2	9.9
Ht	0.37	0.33	0.38	0.37	0.38
Hb (g/L)	131	120	126	121	129
Plt (×10^9^/L)	180	1	218	503	315

TMP/SMX: trimethoprim/sulfamethoxazole; WBC: white blood cell count; Ht: hematocrit; Hb: hemoglobin; Plt: platelets.

**Table 2:** Cases of children with brucellosis and severe thrombocytopenia.

Country	Authors	Year	Number of cases	Treatment	Outcome
Greece	Benecos et al.	1998	1	IVIG, TMP/SMX, streptomycin	Good
	Tsirka et al.	2002	1	IVIG, gentamycin, doxycycline, rifampicin	Good
	Tsolia et al.	2002	2	TMP/SMX, rifampicin	Good

Turkey	Sevinc et al.	2000	1	Corticosteroids, TMP/SMX, rifampicin, ciprofloxacin	Good
	Yalaz et al.	2004	1	Doxycycline, rifampicin	Good
	Ulug et al.	2011	1	TMP/SMX, rifampicin	Good
	Akbayram et al.	2011	5	Doxycycline and rifampicin (>8 years) or TMP/SMX and rifampicin (<8 years)	Good
	Citak et al.	2010	5	IVIG, TMP/SMX, rifampicin	Good
	Aypak et al.	2016	11	Doxycycline and rifampicin (>8 years) or TMP/SMX and rifampicin (<8 years)	Good
	Karaman et al.	2016	15	Doxycycline and rifampicin (>8 years) or TMP/SMX and rifampicin (<8 years)	Good

Saudi Arabia	Benjamin and Annobil	1992	4	Rifampicin, TMP/SMX, doxycycline, streptomycin	Good
	Al-Eissa and Al-Nasser	1993	5	Rifampicin, TMP/SMX, doxycycline, streptomycin	Good
	Benjamin	1995	2	Rifampicin, TMP/SMX, doxycycline	Good

Lebanon	Farah et al.	2010	1	IVIG, TMP/SMX, gentamicin	Good

Israel	Marom et al.	2000	1	Rifampicin, TMP/SMX	Good

Iran	Kamali Aghdam et al.	2016	1	IVIG, rifampicin, TMP/SMX	Good

TMP/SMX: trimethoprim/sulfamethoxazole.
